# Heterozygous *FOXJ1* Mutations Cause Incomplete Ependymal Cell Differentiation and Communicating Hydrocephalus

**DOI:** 10.1007/s10571-023-01398-6

**Published:** 2023-08-24

**Authors:** Connie C. Hou, Danielle Li, Bethany C. Berry, Shaokuan Zheng, Rona S. Carroll, Mark D. Johnson, Hong Wei Yang

**Affiliations:** 1 Department of Neurological Surgery, University of Massachusetts Chan Medical School, 55 Lake Avenue North, Worcester, MA 01655 USA; 2 UMass Memorial Health, Worcester, MA 01655 USA

**Keywords:** FOXJ1, Motile cilia, Hydrocephalus, Ependyma

## Abstract

Heterozygous mutations affecting *FOXJ1*, a transcription factor governing multiciliated cell development, have been associated with obstructive hydrocephalus in humans. However, factors that disrupt multiciliated ependymal cell function often cause communicating hydrocephalus, raising questions about whether *FOXJ1* mutations cause hydrocephalus primarily by blocking cerebrospinal fluid (CSF) flow or by different mechanisms. Here, we show that heterozygous *FOXJ1* mutations are also associated with communicating hydrocephalus in humans and cause communicating hydrocephalus in mice. Disruption of one *Foxj1* allele in mice leads to incomplete ependymal cell differentiation and communicating hydrocephalus. Mature ependymal cell number and motile cilia number are decreased, and 12% of motile cilia display abnormal axonemes. We observed decreased microtubule attachment to basal bodies, random localization and orientation of basal body patches, loss of planar cell polarity, and a disruption of unidirectional CSF flow. Thus, heterozygous *FOXJ1* mutations impair ventricular multiciliated cell differentiation, thereby causing communicating hydrocephalus. CSF flow obstruction may develop secondarily in some patients harboring *FOXJ1* mutations.

**Graphical Abstract:**

Heterozygous *FOXJ1* mutations impair motile cilia structure and basal body alignment, thereby disrupting CSF flow dynamics and causing communicating hydrocephalus.

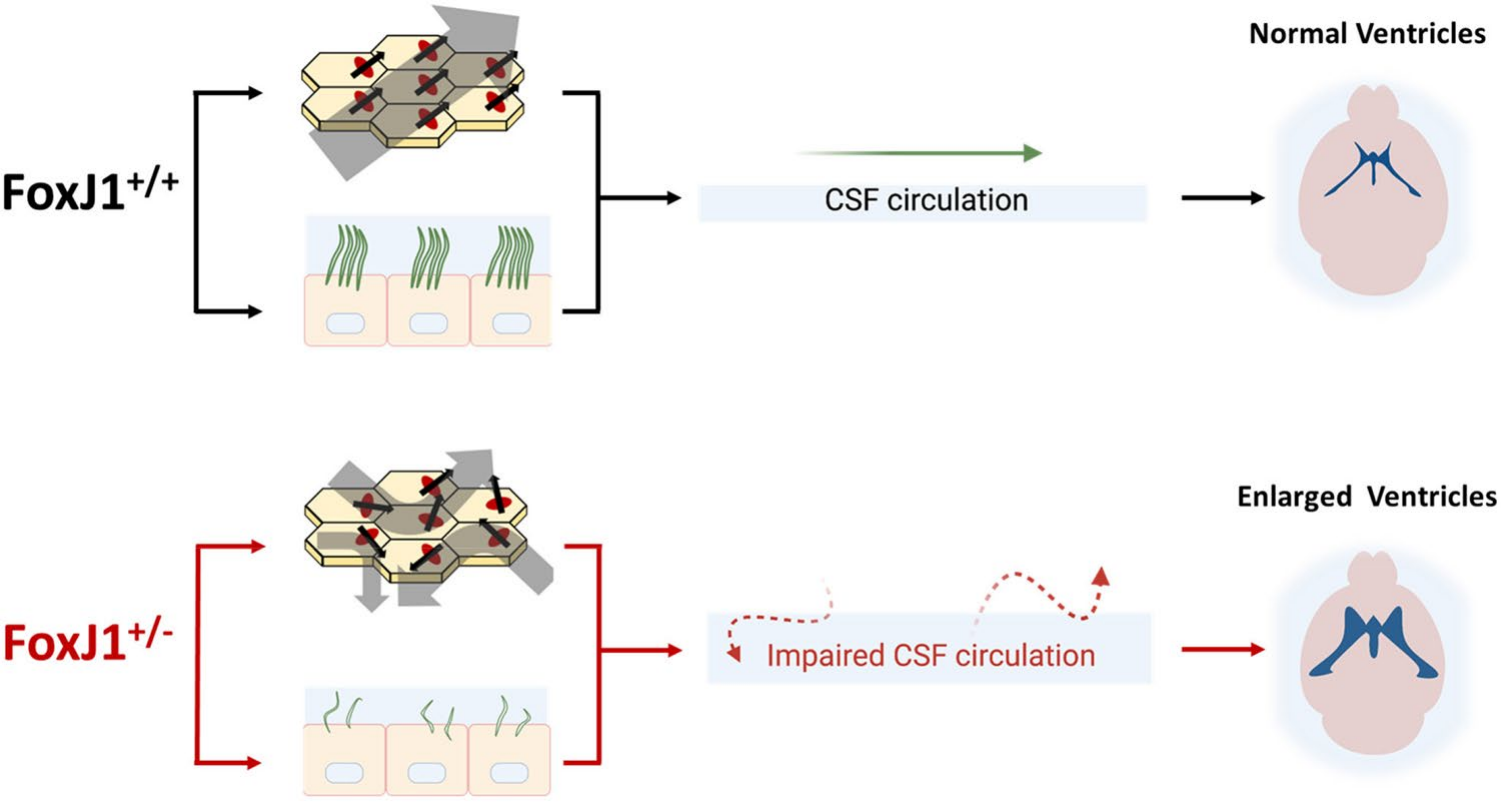

**Supplementary Information:**

The online version contains supplementary material available at 10.1007/s10571-023-01398-6.

## Background

Hydrocephalus is a family of disorders that is defined by the abnormal accumulation of cerebrospinal fluid (CSF) in the brain. It can occur spontaneously at birth (e.g., congenital hydrocephalus), spontaneously during adulthood (e.g., idiopathic normal pressure hydrocephalus), or after nervous system insults such as hemorrhage, trauma, infection, or neoplastic growth. Overall, hydrocephalus is a relatively common disorder that represents a significant health burden. The global incidence of congenital hydrocephalus ranges from 79 per 100,000 births in high-income countries to 123 per 100,000 births in low- and middle-income countries (Dewan et al. 2018). Idiopathic normal pressure hydrocephalus is a form of adult-onset communicating hydrocephalus that has been estimated to affect more than 5% of the population over the age of 75 (Martin-Laez et al. 2015). The recent identification of mutations associated with congenital hydrocephalus and idiopathic normal pressure hydrocephalus has provided new insights into the mechanisms underlying these disorders (Shaheen et al. 2017; Jin et al. 2020; Yang et al. 2021; Morimoto et al. 2019). Included among these mechanisms are defects in neuroprogenitor cell/cortical development, loss of cell polarity, abnormalities of cell–cell adhesion, obstruction to CSF flow, and ciliopathies (Jin et al. 2020; Duy et al. 2022; Ross et al. 2005; Wallmeier et al. 2022; Morimoto et al. 2019). Whereas many of the mutations responsible for hydrocephalus have an autosomal recessive pattern of inheritance, some display X-linked (*L1CAM, AP1S2*) (Okamoto et al. 1996; Saillour et al. 2007) or autosomal-dominant (*CFAP43*, *FOXJ1*) inheritance patterns (Wallmeier et al. 2019; Shapiro et al. 2021; Morimoto et al. 2019).

*FOXJ1* encodes a transcription factor that is essential for the development of epithelial cells with motile cilia that create the directional fluid flow necessary to establish left–right asymmetry and mucociliary clearance in the respiratory tract. Homozygous or heterozygous deletion of *FOXJ1* leads to situs inversus and mucociliary clearance dysfunction in humans and mice (Brody et al. 2000; Muthusamy et al. 2014). Several reports have identified heterozygous *FOXJ1* mutations in association with obstructive hydrocephalus in humans (Wallmeier et al. 2019; Shapiro et al. 2021; Jin et al. 2020). However, factors that disrupt the function of motile cilia often cause communicating hydrocephalus with no evidence of obstruction to CSF flow through the ventricular system, although the exact mechanisms by which this occurs are poorly understood. Thus, it is unclear whether the primary mechanism by which heterozygous mutation of *FOXJ1* causes hydrocephalus is by blocking CSF flow, or whether other mechanisms are responsible. We report here that heterozygous *FOXJ1* mutation is also associated with communicating hydrocephalus in humans, and *FOXJ1* haploinsufficiency in mice leads to incomplete differentiation of multiciliated ependymal cells and communicating hydrocephalus with no evidence of CSF flow obstruction.

## Materials and Methods

### Clinical Information

Clinical evaluation of patients with idiopathic normal pressure hydrocephalus and whole exome analysis of patient DNA were performed after written informed consent had been obtained under the auspices of an IRB-approved human subjects protocol as we have described previously (Yang et al. 2021).

### Animals

All experiments and procedures involving mice were approved by the University of Massachusetts Chan Medical School Institutional Animal Care and Use Committee (IACUC). The *Foxj1*^*WT/CreERT2::GFP*^ mouse line was obtained from the Jackson Laboratory (JAX stock #027012) and was bred to produce both *Foxj1*^*WT/CreERT2::GFP*^ and *Foxj1*^*WT/WT*^ mice. All mice were genotyped according to the protocol from the Jackson Laboratory (https://www.jax.org/Protocol?stockNumber=027012&protocolID=17668).

### MRI

T2-weighted MR images of the brains of *Foxj1*^*WT/WT*^ and heterozygous *Foxj1*^*WT/CreERT2::GFP*^ mice at 4 weeks old were obtained using a Bruker BioSpec 70/30 7 T MRI in The Advanced MRI Center at UMass Chan Medical School. The mice were placed under anesthesia using isoflurane at 5% for induction and 1–2% for maintenance, and this was mixed with oxygen flowing at 2 L per minute. The respiratory rate was maintained at 30 per minute. Ventricular volume was quantified using the NIH ImageJ software and a custom automated computer algorithm.

### Intraventricular Injection of Fluorescent Dextran to Trace CSF Circulation

Mice were anesthetized by intraperitoneal injection of xylazine (10 mg/kg) and ketamine (100 mg/kg), and adequate anesthesia was confirmed before surgery by pinching the footpad. A 31G injection needle was positioned 0.5 mm posterior and 1.1 mm lateral to bregma, and then advanced 2.5 mm below the skull surface. Approximately 2 μl of 70 kDa fluorescent dextran (10 mg/ml in saline) was slowly injected into the lateral ventricle over a 3-min period. This was followed by a 2-min waiting period to allow the dextran to circulate. The mice were then euthanized using CO_2_, and the brains were harvested and fixed in 4% paraformaldehyde overnight. The brains were then submerged in a 15% sucrose solution followed by a 30% sucrose solution, and sagittal cryosections through the aqueduct were prepared for fluorescent imaging with counterstaining of nuclei using DAPI.

### Western Blot

Mouse brains were harvested, and the ventricular zone (~ 1 mm in thickness) was microdissected using a micro knife. The tissues were lysed using RIPA buffer (BP-115, Boston Bioproducts, USA) containing proteinase inhibitor and phosphatase inhibitor. Protein content in the lysates was quantified using the Pierce BCA protein assay kit (ThermoFisher Scientific) according to the manufacturer’s instructions. Lysates were separated using gel electrophoresis (SDS-PAGE) and analyzed by Western blot. The following antibodies were used: anti–FOXJ1 (14–9965-82, 1:500, Invitrogen, USA), anti–β-Actin (4970S, 1:1000, Cell Signaling Technology, USA), and an anti-rabbit secondary antibody. Immunostained bands were detected using the chemiluminescent method (Pierce ECL Western Blotting Substrate; ThermoFisher Scientific), and images were obtained using a KODAK Image Station 4000 System.

### TaqMan Real-Time PCR

Total RNA was extracted from cell lines with TRIzol (Invitrogen, Carlsbad, CA) according to the manufacturer's protocol. Randomly primed cDNA was prepared using 1 μg of total RNA from each sample and SuperScript IV Reverse Transcriptase. 18 ng of each cDNA was then used for real-time PCR analysis in a final reaction volume of 20 μl. PCR probes for *β-actin* (Mm00607939_s1) and mouse *Foxj1* (Mm01267279_m1) were purchased from ThermoFisher Scientific. Samples were analyzed in triplicate using the ABI 7300 software system (Applied Biosystems, Foster City, CA) with ΔΔCt quantification. The experiments were performed in triplicate, and statistical analysis was performed using an unpaired *t*-test.

### Immunohistochemical Staining

Brains from 4 to 6-week-old mice were harvested and thin slices of the ventricular walls were then microdissected and fixed in 4% paraformaldehyde (BM-155, Boston Bioproducts, USA). These slices were stained for fluorescence immunohistochemistry using antibodies directed against acetylated α-tubulin (sc-23950, Lot# L1019, 1:500, Santa Cruz Biotechnology, USA), γ-tubulin (NB500-574, Lot#542972020916G, 1:500, Novus, USA), VANGL2 (sc-515187, Lot# C0520, 1:100, Santa Cruz Biotechnology, USA; MABN750 clone 2G4, Lot#3,794,319, MilliporeSigma, USA), S100β (PA5-78,161, Lot#137187E, 1:500, Invitrogen, USA) FGFR1OP (FOP) (H00011116-M01, Lot#N2-131-2B1, Abnova, USA), alpha smooth muscle actin (αSMA) (48,938, Clone 1A4, Lot#2, 1:100, Cell Signaling Technology, USA), FOXJ1 (14–9965-82, Lot#2,488,476, 1:200, Invitrogen, USA), or GFAP (13–0300, Clone 2.2B10, Lot#TJ275318, 1:500, Invitrogen, USA). Tight junctions were stained using an antibody directed against ZO-1 (339,188, Clone 1A12, Lot#XC345409, 1:500, Invitrogen, USA). The Alexa Fluor labeled secondary antibodies were purchased from ThermoFisher Scientific. Immunoreactivity was imaged using laser confocal fluorescence microscopy. All experiments were repeated at least twice.

### Planar Cell Polarity and Rotational Polarity Analysis

Analysis of planar cell polarity was performed using images of the lateral ventricular surface that had been stained for ZO-1, γ-tubulin, or Vangl2 immunoreactivity to visualize tight junctions at cell boundaries, basal bodies (BB), or planar membrane polarization, respectively. Identification of cell membrane boundaries and basal body patch location and orientation was completed manually using the NIH ImageJ software. The centroids of cells and basal bodies, and Feret’s angles were calculated using ImageJ. Translational distance and BB patch angle were calculated using a custom macro script (Fig. 5d) (https://github.com/conniehou3/MCC-BB-Polarity-Analysis). Analysis of rotational polarity was performed using images of the lateral ventricular surface stained for FOP and γ-tubulin immunoreactivity to visualize the relationship between basal feet and basal bodies. Vectors were drawn (black arrows) from each FOP-positive area to the nearest γ-tubulin-positive area to indicate the rotational orientation of each basal body, i.e., the rotational polarity (Fig. 5b) (Ryu et al. 2021).

### Ependymal Flow Analysis

To analyze ependymal flow, fluorescent microspheres were applied to the surface of the lateral ventricle in ex vivo brain slices (Fig. 6a, 6b, Supplementary Videos) (Sawamoto et al. 2006; Mirzadeh et al. 2010). Mice were euthanized with CO_2_. The brains were then harvested and sectioned through the lateral ventricle in the coronal plane at a thickness of 1 mm. The sections of the lateral ventricle were then immobilized on a clean dissecting dish using cyanoacrylate glue and submerged in PBS at pH 7.4. Approximately 0.3 µl of fluorescent microspheres prediluted 1:5000 in PBS were dispersed into the lateral ventricle lumen. The movement of the microspheres was recorded by video microscopy and the direction of movement of individual microspheres was analyzed using ImageJ (see Supplementary videos). Statistical significance was determined using Watson’s Two-Sample Test of Homogeneity.

### Scanning and Transmission Electron Microscopy

For transmission electron microscopy (TEM), mouse brain lateral ventricles were harvested, cut into 1 mm^3^ slices, and immediately fixed in 2.5% glutaraldehyde/1.6% paraformaldehyde in 0.1 M Sodium Cacodylate buffer pH 7.2 and left overnight at 4 °C. The samples were then rinsed three times in the same fixation buffer and post-fixed with 1% osmium tetroxide for 1 h at room temperature before rinsing three times with DH_2_O for 10 min. Next, samples were stained *en bloc* with 1% uranyl acetate for 30 min at room temperature and dehydrated through a graded ethanol series (10, 30, 50, 70, 85, 95%, and 3 × 100% ethanol). Specimens were infiltrated first with two changes of 100% propylene oxide before leaving them overnight in a 50%/50% propylene oxide/SPI-Pon 812 resin mixture. Over the following two days, six changes of fresh 100% SPI-Pon 812 resin were done before the samples were polymerized at 68 °C in flat molds. The samples were then reoriented for horizontal sections. The thin sections (approx. 70 nm) were placed on gold support grids and contrasted with Lead citrate and Uranyl acetate. Sections were examined using the CM10 transmission electron microscope with 80 kV accelerating voltage, and images were captured using a Gatan TEM CCD camera.

For scanning electron microscopy (SEM), mouse brain lateral ventricles were harvested and fixed immediately by immersion in 2.5% glutaraldehyde/1.6% paraformaldehyde in 0.1 M Sodium Cacodylate buffer (pH 7.2) and left overnight at 4 °C. The fixed samples were then washed three times in the same fixation buffer. After the third wash, the brains were dehydrated through a graded series of ethanol to 100%, transferred into porous sample holders, and critical point-dried in liquid CO_2_. After being dried in CO_2_, the samples were transferred to aluminum SEM stubs coated with adhesive carbon tape. The edges were painted with silver conductive paste and the SEM stubs were sputter-coated with Au/Pd (80/20). The specimens were then examined using an FEI Quanta 200 FEG MKII scanning electron microscope at 10 kV accelerating voltage.

All scanning and transmission electron microscopy were carried out at the UMass Chan Medical School Core Electron Microscopy Facility with support in part by Award Numbers S10OD025113-01 and S10OD021580 from the National Center for Research Resources.

### Statistical Analysis

Across all experiments, quantified values are presented as mean ± standard error of mean (SEM). Statistical significance was calculated using the appropriate t-test or Watson’s Two-Sample Test of Homogeneity. Differences between means were considered statistically significant when *p* < 0.05.

## Results

### A Heterozygous Pathogenic *FOXJ1* Mutation in a Patient with Normal Pressure Hydrocephalus

**Fig. 1:**
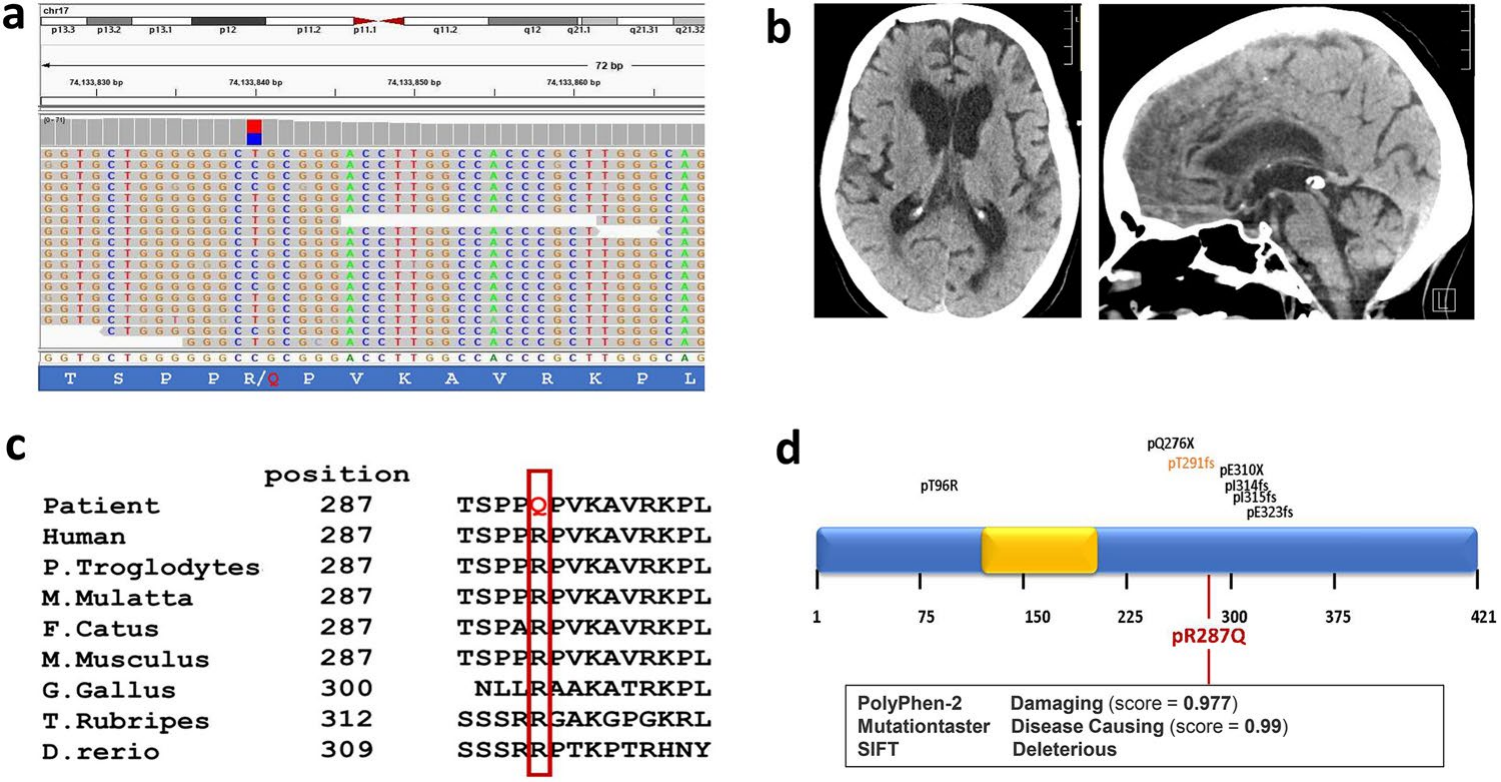
Heterozygous *FOXJ1* mutation in a patient with communicating hydrocephalus. **a** Whole exome sequencing of DNA from a patient with idiopathic normal pressure hydrocephalus revealed a heterozygous mutation of FOXJ1:exon3:c.C860T:p.R287Q, Chr 17: 74,133,840. **b** Axial and sagittal non-contrast head CT images showing enlargement of the lateral, third, and fourth ventricles as well as a patent cerebral aqueduct in a patient with idiopathic normal pressure hydrocephalus (iNPH) and a heterozygous FOXJ1 mutation. **c** Amino acid R287 in FOXJ1 is highly conserved throughout many vertebrate species (red rectangle). **d** A summary of published pathogenic *FOXJ1* variants found in humans with hydrocephalus. The variants highlighted in black and orange occurred in the context of obstructive hydrocephalus (Wallmeier et al. 2019; Shapiro et al. 2021; Jin et al. 2020). The variant in orange was diagnosed in a 54-year-old patient who had a patent cerebral aqueduct and dilated lateral, third, and fourth ventricles. This was postulated by the authors to be due to closure of the Foramen of Magendie and the Foramina of Luschka. The p.R287Q mutation in the *FOXJ1* variant highlighted in red was diagnosed in a patient with communicating normal pressure hydrocephalus described in the current report. This mutation is predicted to impair protein function by multiple computer algorithms

In an effort to identify genetic factors that contribute to adult-onset idiopathic normal pressure hydrocephalus (iNPH), we performed whole exome sequencing of DNA obtained from 53 patients with shunt-responsive iNPH (Yang et al. 2021). In one of the 53 iNPH patients, we identified a heterozygous mutation affecting *FOXJ1* (exon3:c.C860T:p.R287Q, Chr 17: 74,133,840) (Fig. 1a). Imaging of the brain in this patient revealed a patent cerebral aqueduct as well as enlargement of the lateral, third, and fourth ventricles, a pattern suggestive of communicating hydrocephalus (Fig. 1b). The patient underwent several days of lumbar CSF drainage during which 5–15 cc of CSF were drained each hour, providing functional evidence that there was no obstruction to CSF flow. This patient was diagnosed with normal pressure hydrocephalus at 70 years old and underwent a shunting procedure. Her medical history was also notable for COPD and non-small cell lung cancer, for which she underwent chemotherapy. The exon3:c.C860T:p.R287Q mutation in *FOXJ1* has a minor allele frequency (MAF) of 2.97e-5 in the general population (https://gnomad.broadinstitute.org/) and 0.0094 among iNPH patients (*p* < 0.0001, *X*^2^ Test with Yates correction). In the FOXJ1 protein, amino acid R287 is evolutionarily conserved from zebrafish to humans (Fig. 1c), suggesting that it is functionally important. In support of this notion, three independent computer algorithms (PolyPhen-2, SIFT and Mutation Taster) predicted that the R287Q mutation is functionally damaging and disease causing (Fig. 1d). A review of previously reported *FOXJ1* mutations linked to hydrocephalus revealed that nearly all of the mutations are clustered near amino acid 300 (Fig. 1d) (Shapiro et al. 2021; Wallmeier et al. 2019; Jin et al. 2020). This region is listed in the Uniprot database as a specific region of FOXJ1 mediating protein–protein interactions (https://www.uniprot.org/uniprotkb/Q92949/entry).

### Heterozygous *Foxj1* Deletion Decreases FOXJ1 Expression and Causes Communicating Hydrocephalus

**Fig. 2:**
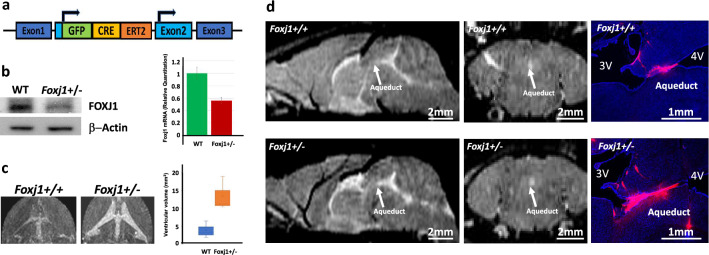
Heterozygous *Foxj1* mutation decreases FOXJ1 expression and causes communicating hydrocephalus. **a** Schematic diagram depicting the insertion of CreERT2::GFP into Exon 2 of the *Foxj1* gene. This construct was used to generate *Foxj1* mutant mice. **b** Western blot and TaqMan real-time PCR analyses comparing FOXJ1 protein and mRNA expression in WT (*Foxj1*^*+/+*^) mice versus *Foxj1*^*WT/CreERT2::GFP*^* mice* (*Foxj1*^*+/-*^). β-actin was used as a protein loading control. **c** MR images of the brains of WT (*n* = 3) and *Foxj1*^*WT/CreERT2::GFP*^ mice (*n* = 3). Note the enlarged ventricles in *Foxj1*^*WT/CreERT2::GFP*^ mice. **d** Sagittal and coronal sections of the brain revealed a patent cerebral aqueduct. Injection of 70 kDa dextran into the ventricles confirmed CSF flow through the aqueduct in *Foxj1*^*WT/CreERT2::GFP*^ mice

Previous reports indicated that mice with biallelic insertion of CreERT2::GFP (CEG) into Exon 2 of *Foxj1* (Fig. 2a) showed a *Foxj1-null* phenotype, indicating that the insertion disrupts the *Foxj1* gene (Muthusamy et al. 2014). Unlike the robust phenotype observed in mice with homozygous disruption of *Foxj1*, however, heterozygous *Foxj1*^*WT/CreERT2::GFP*^ mice were considered to be relatively normal when compared to wild-type (WT) mice (Muthusamy et al. 2014; Abdi et al. 2018; Brody et al. 2000). To examine the impact of heterozygous *Foxj1* deletion more closely, we explored the effect of disrupting one *Foxj1* allele on FOXJ1 protein expression in *Foxj1*^*WT/CreERT2::GFP*^ mice. Total protein and RNA were extracted from tissue that was microdissected from the lateral ventricular walls of *Foxj1*^*WT/CreERT2::GFP*^ and *Foxj1*^*WT/WT*^ mice and subsequently examined for FOXJ1 protein expression by Western blot and for mRNA expression by TaqMan real-time PCR. Decreased FOXJ1 protein and mRNA were detected in *Foxj1*^*WT/CreERT2::GFP*^ mice compared to *Foxj1*^*WT/WT*^ mice (Fig. 2b).

Although de novo heterozygous *FOXJ1* mutation is associated with congenital obstructive hydrocephalus in humans (Jin et al. 2020; Wallmeier et al. 2019; Shapiro et al. 2021), mice with heterozygous *Foxj1* mutations did not display the domed head and perinatal death that is frequently associated with congenital obstructive hydrocephalus in mice. Unlike homozygous *Foxj1* knockout mice that have a shortened lifespan of a few weeks, *Foxj1*^*WT/CreERT2::GFP*^ mice had a normal lifespan and appeared grossly normal (Brody et al. 2000). We examined the cerebral ventricles of *Foxj1*^*WT/CreERT2::GFP*^ mice (*n* = 3) using T2-weighted magnetic resonance imaging (MRI) and found that the ventricular volume was moderately (but significantly) enlarged when compared to the ventricular volume of *Foxj1*^*WT/WT*^ mice (*n* = 3, Fig. 2c), suggesting that heterozygous *Foxj1* deletion leads to *Foxj1* haploinsufficiency and hydrocephalus in mice. Importantly, the degree of hydrocephalus in *Foxj1*^*WT/CreERT2::GFP*^ heterozygotes was less than that observed in homozygous *Foxj1* mutant mice (Brody et al. 2000; Muthusamy et al. 2014). Coronal and sagittal MR images indicated that the cerebral aqueduct remained patent in heterozygous *Foxj1*^*WT/CreERT2::GFP*^ animals (Fig. 2d). Injection of fluorescent dextran (70 kDa) into the lateral ventricle of *Foxj1*^*WT/CreERT2::GFP*^ and WT mice resulted in rapid circulation of fluorescent dextran throughout the ventricular system and subarachnoid space, confirming patency of the aqueduct (Fig. 2d). Together, these results indicated that haploinsufficiency of *Foxj1* causes communicating hydrocephalus in mice.

### Heterozygous *Foxj1* Deletion Causes Incomplete Differentiation of Ependymal Cells

**Fig. 3:**
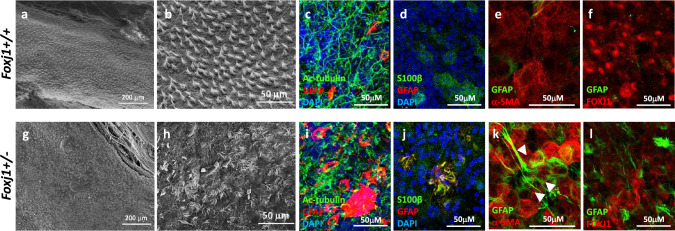
Heterozygous* Foxj1* mutation causes incomplete differentiation of ependymal cells. Lower magnification **(a, g)** and higher magnification **(b, h)** scanning electron microscopy images of the brain lateral ventricular surface of WT (*Foxj1*^*+/+*^) and *Foxj1*^*WT/CreERT2::GFP*^ (*Foxj1*^*+/-*^) mice. **c, i** Immunohistochemistry of the lateral ventricular wall revealed areas that lacked acetylated-tubulin (Ac-tubulin) immunoreactivity (a marker for motile cilia) in *Foxj1*^*WT/CreERT2::GFP*^ mice when compared to WT mice. Each of the areas lacking motile cilia contained clusters of cells expressing glial fibrillary acidic protein (GFAP), a marker for radial glia, and astrocytes. **d, j** Clusters of cells expressing both GFAP and the ependymal cell marker, S100β, were observed on the surface of the lateral ventricles of heterozygous *Foxj1*^*WT/CreERT2::GFP*^ mice but not WT mice. Immunohistochemical staining showed colocalization of GFAP with αSMA **(e, k)** and FOXJ1 **(f, l)**, ependymal cell markers, on the ventricular surface of *Foxj1*^*WT/CreERT2::GFP*^ mice that was not observed in WT mice

*Foxj1* regulates ciliogenesis (Stubbs et al. 2008; Abdi et al. 2018) and is required to maintain the multiciliated phenotype of ependymal cells (Abdi et al. 2018). Ependymal motile cilia dysfunction impairs normal CSF circulation and causes hydrocephalus, although the exact mechanism by which this occurs is unclear (Kumar et al. 2021; Olstad et al. 2019). To examine the effects of heterozygous* Foxj1* deletion on ependymal cells, we used scanning electron microscopy (SEM) to examine the surface of the lateral ventricular wall of *Foxj1*^*WT/CreERT2::GFP*^ mice and WT mice. Although numerous multiciliated ependymal cells were present throughout the lateral ventricular surface in  WT and heterozygous *Foxj1*^*WT/CreERT2::GFP*^ mice (Fig. 3a, 3g), there were numerous areas on the lateral ventricular wall of *Foxj1*^*WT/CreERT2::GFP*^ mice that lacked multiciliated ependymal cells when compared to WT mice (Fig. 3b, 3h).

A previous study indicated the presence of glial fibrillary acidic protein (GFAP), a marker of both radial glia and astrocytes, in developing ependymal cells and its absence in mature ependymal cells (Roessmann et al. 1980). It was proposed that either the synthesis of GFAP occurs only at an early stage of ependymal cell maturation, or that the intermediate filaments assembled in developing ependymal cells are antigenically distinct from those of the mature cells (Roessmann et al. 1980). Immunohistochemistry indicated that each of the areas lacking ciliated ependymal cells in heterozygous *Foxj1*^*WT/CreERT2::GFP*^ mice contained numerous cells expressing GFAP (Fig. 3c, 3i). Numerous cells strongly expressing GFAP were randomly distributed throughout the lateral ventricular wall of Foxj1^WT/CreERT2::GFP^ mice, but were not observed in WT mice (Supplementary Fig. 1). Importantly, many of the GFAP + cells lacking motile cilia expressed the ependymal cell marker, S100β. Moreover, an increased number of multiciliated cells lining the ventricular surface expressed GFAP and S100β in heterozygous *Foxj1*^*WT/CreERT2::GFP*^ mice when compared to WT mice (Fig. 3d, 3j). Because S100β + ependymal cells are derived from GFAP + radial glia (Wu et al. 2020), these data suggest incomplete differentiation of ependymal cells in *Foxj1* heterozygous mice. Incomplete differentiation of ependymal cells in heterozygous *Foxj1*^*WT/CreERT2::GFP*^ mice was further confirmed using immunohistochemistry for two additional ependymal cell markers, smooth muscle actin alpha (αSMA)(Herranz-Perez et al. 2022; Shah et al. 2018) and FOXJ1 itself. We observed increased colocalization of GFAP with αSMA and with FOXJ1 in heterozygous *Foxj1*^*WT/CreERT2::GFP*^ mice when compared to WT mice (Fig. 3e, 3f, 3k, 3l).

### Heterozygous *Foxj1* Deletion Causes Ependymal Motile Cilia Abnormalities

**Fig. 4:**
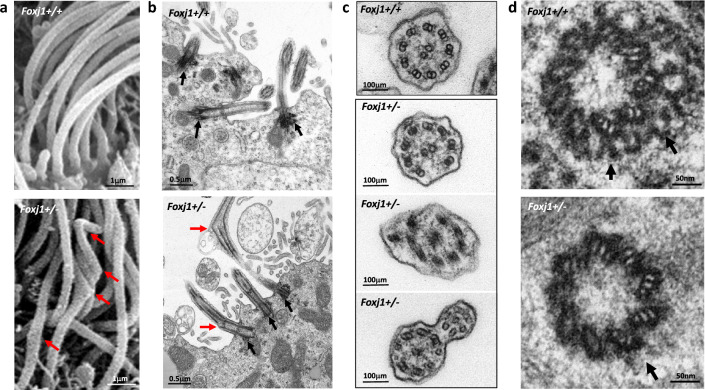
Heterozygous *Foxj1* mutation causes ependymal motile cilia abnormalities. **a** Scanning electron microscopy (SEM) images of motile cilia bundles on ependymal cells displaying bulbous dilation and sharp angulation of a subset of cilia axonemes (red arrows) in *Foxj1*^*WT/CreERT2::GFP*^ (*Foxj1*^*+/-*^) mice, but not WT (*Foxj1*^*+/+*^) mice. **b** Transmission electron microscopy (TEM) of the ventricular surface of WT mice (upper panel) or *Foxj1*^*WT/CreERT2::GFP*^ mice. Note the bulbous dilation and angulation of a subset of motile cilia axonemes as well as decreased density of basal bodies at the base of the cilia (black arrows) in *Foxj1*^*WT/CreERT2::GFP*^ mice when compared to WT mice. **c** Cross-section transmission electron microscopy (TEM) images showed defects in the normal 9 + 2 microtubule structure of motile cilia in *Foxj1*^*WT/CreERT2::GFP*^ mice when compared to WT mice. **d** TEM images showing basal bodies at the base of motile cilia with decreased cytoskeletal attachments (black arrows) in *Foxj1*^*WT/CreERT2::GFP*^ mice when compared to WT mice

Although SEM of the surface of the lateral ventricle of *Foxj1*^*WT/CreERT2::GFP*^ mice revealed the presence of numerous multiciliated ependymal cells, we observed a noticeable decrease in the number of motile cilia per cell when compared to WT mice (Fig. 3b, 3h). High-magnification SEM images indicated that the axonemes of ependymal motile cilia in *Foxj1*^WT/WT^ mice were smooth in contour. In contrast, a subpopulation of ependymal motile cilia axonemes in *Foxj1*^*WT/CreERT2::GFP*^ mice displayed abnormal localized swellings or sharply angled fracture-like bends (Fig. 4a, 4b).

We used transmission electron microscopy (TEM) to obtain ultrastructural information about ependymal motile cilia in these mice. Whereas a majority of ependymal motile cilia in *Foxj1*^*WT/CreERT2::GFP*^ mice displayed a normal 9 + 2 axoneme structure, about 12% displayed absent or mislocalized microtubule doublets, leading to an incomplete outer ring of the usual 9 + 2 microtubule doublet structure with associated abnormal flattening or outpouching of the cilia membrane at the affected sites. Fusion of membranes between cilia, some of which displayed an abnormal 9 + 2 structure, was frequently observed in *Foxj1*^*WT/CreERT2::GFP*^ mice, but not in *Foxj1*^WT/WT^ mice (Fig. 4c). In addition, the base of ependymal motile cilia in *Foxj1*^*WT/CreERT2::GFP*^ mice lacked the lateral cytoskeletal attachments that were commonly observed in *Foxj1*^WT/WT^ mice (Fig. 4b, 4d).

### Heterozygous *Foxj1* Deletion Disrupts Planar Cell Polarity

**Fig. 5:**
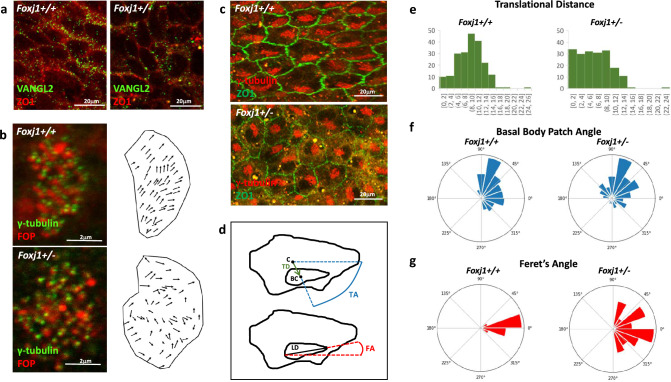
Heterozygous *Foxj1* mutation disrupts translational and planar cell polarity. **a** Fluorescence immunohistochemistry for the tight junction protein, ZO-1 (red), and the planar cell polarity protein, VANGL2 (green), in ependymal cells on the lateral ventricular surface of *WT (Foxj1*^*+/+*^ and *Foxj1*^*WT/CreERT2::GFP*^ (*Foxj1*^*+/-*^) mice. Note the directionally asymmetric membrane association of VANGL2 immunoreactivity in ependymal cells from WT but not *Foxj1*^*WT/CreERT2::GFP*^ mice. **b** Immunohistochemistry for basal foot marker FOP (red) and the basal body marker, γ-tubulin (green) in ependymal cells on the lateral ventricular surface of WT, and *Foxj1*^*WT/CreERT2::GFP*^ mice. The rotational polarities are indicated by vectors (black arrows) connecting FOP-positive areas to the nearest γ-tubulin-positive areas. **c** Immunohistochemistry for ZO-1 (green) and the basal body marker γ-tubulin (red) in ependymal cells on the lateral ventricular surface of WT and *Foxj1*^*WT/CreERT2::GFP*^ mice. Basal body clusters are directionally oriented and asymmetrically displaced from the cell center in a consistent manner in WT ependymal cells but not in *Foxj1*^*WT/CreERT2::GFP*^ ependymal cells. **d** Schematic describing how translation distance (TD) and BB patch angle (TA) were calculated from the cell centroid (C) and basal body centroid (BC), and how Feret’s angle (FA) was calculated by using the longest diameter (LD) of each basal body. **e** Bar graphs illustrating the distribution of translational distance in ependymal cells on the lateral ventricular surface of WT and *Foxj1*^*WT/CreERT2::GFP*^ mice. The translational distance of ependymal cells in *Foxj1*^*WT/CreERT2::GFP*^ mice was significantly decreased when compared to that of *Foxj1*^*WT/WT*^ mice (two tailed *t*-test, *p* < 0.001). **f** Circular bar plots displaying the distribution of BB patch angles of ependymal cells on the lateral ventricular surface of *Foxj1*^*WT/CreERT2::GFP*^ and WT mice. **g** Circular bar plots displaying the distribution of Feret’s angles in ependymal cells on the lateral ventricular surface of *Foxj1*^*WT/CreERT2::GFP*^ and WT mice

Directional CSF flow caused by the coordinated and organized beating of motile cilia is dependent upon the establishment of planar cell polarity (PCP) (Takagishi et al. 2017). Immunohistochemistry for VANGL2, a key regulator of planar cell polarity, showed the characteristic asymmetric, membrane-associated alignment of immunoreactivity indicative of planar cell polarity in ependymal cells of *Foxj1*^*WT/WT*^ mice. In contrast, VANGL2 immunoreactivity in *Foxj1*^*WT/CreERT2::GFP*^ mice was diffusely distributed in ependymal cells (Fig. 5a).

Previous reports indicated that ependymal cells must maintain a planar organization of their cilia to generate unidirectional CSF flow (Kishimoto and Sawamoto 2012; Ohata et al. 2014). The rotational alignment of basal bodies (BBs) is key to the coordinated beating of motile cilia (Hirota et al. 2010). Defects in the coordinated beating of ependymal cell cilia lead to abnormal CSF accumulation and hydrocephalus. Each cilium originates from a basal body that has a basal foot protruding from one side. A uniform alignment of these basal body feet is crucial for the coordination of cilia movement (Ryu et al. 2021). The FGFR1 oncogene partner (FOP) is a marker for the basal foot of motile cilia, and γ-tubulin is a marker for the basal body. We performed immunohistochemistry for FOP and γ-tubulin to analyze the rotational polarity of cilia basal bodies in the mouse lateral ventricle. A vector (black arrows) connecting each FOP-positive area to the nearest γ-tubulin-positive area indicates the rotational polarity of each basal body (Ryu et al. 2021). The basal body rotational polarity in ependymal cells of *Foxj1*^*WT/WT*^ mice displayed a unidirectional orientation. In contrast, basal body rotational polarity in ependymal cells of heterozygous *Foxj1*^*WT/CreERT2::GFP*^ mice were randomly oriented (Fig. 5b). We also analyzed planar cell polarity by examining the intracellular localization and orientation of basal body patches located at the base of the motile cilia bundles using γ-tubulin immunohistochemistry. The basal body patches of ependymal cells in *Foxj1*^*WT/WT*^ mice displayed consistency in their directional orientation and in their intracellular localization relative to the centroid of each cell. In contrast, basal body patches in ependymal cells of heterozygous *Foxj1*^*WT/CreERT2::GFP*^ mice were located more centrally within cells and were randomly oriented (Fig. 5c). We calculated translational polarity (the distance between the centroid of the cells and basal body patches), BB patch angle, and Feret’s angle (the degree of basal body patch rotation with respect to the horizontal axis (Fig. 5d). The translational distance of ependymal cells in *Foxj1*^*WT/CreERT2::GFP*^ mice was significantly decreased compared to *Foxj1*^*WT/WT*^ mice (*p* < 0.05, Fig. 5e). The BB patch angle and Feret’s angle in ependymal cells from *Foxj1*^*WT/CreERT2::GFP*^ mice were distributed across a larger range when compared to that of *Foxj1*^*WT/WT*^ mice (Fig. 5f, 5g). In total, we analyzed 207 and 191 cells of the ventricular wall in *Foxj1*^*WT/WT*^ and *Foxj1*^*WT/CreERT2::GFP*^ mice, respectively. Taken together, these data indicate that haploinsufficiency of *Foxj1* impairs planar cell polarity and the coordinated orientation of motile cilia bundles in ependymal cells.

### Heterozygous *Foxj1* deletion alters CSF flow dynamics

**Fig. 6:**
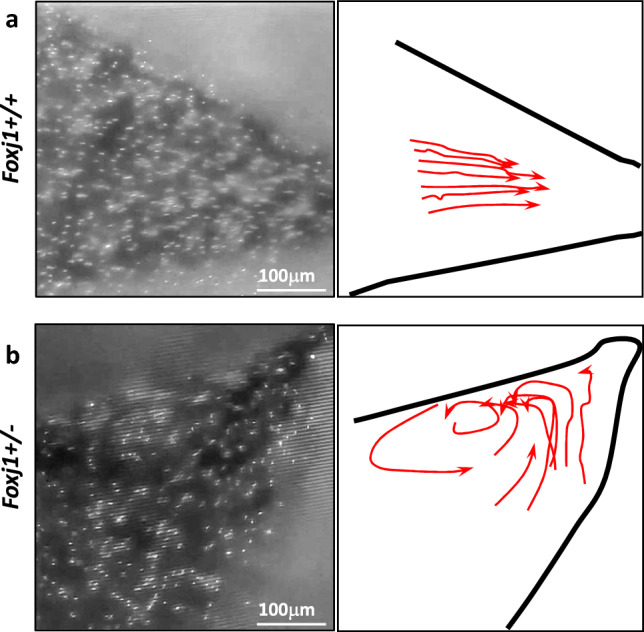
Heterozygous *Foxj1* deletion alters CSF flow dynamics. **a** Screen capture from a video recording of the movement of fluorescent microspheres deposited inside the lateral ventricle of WT (*Foxj1*^*+/+*^) mice. Analysis of CSF flow direction was performed by following changes in microsphere location over time. **b** Screen capture from a video recording of fluorescent microspheres deposited inside the lateral ventricle of heterozygous *Foxj1*^*WT/CreERT2::GFP*^ (*Foxj1*^*+/-*^) mice. Analysis of CSF flow direction was performed by following changes in microsphere location over time

Published reports indicate that ependymal motile cilia dysfunction impairs normal CSF circulation and leads to hydrocephalus (Ji et al. 2022). To determine whether heterozygous *Foxj1* deletion alters cilia-mediated CSF flow dynamics, we applied fluorescent microspheres to acutely harvested brain explants containing a coronal cross section of the lateral ventricles (*Foxj1*^*WT/WT*^ (*n* = 2); *Foxj1*^*WT/CreERT2::GFP*^ (*n* = 2)). In *Foxj1*^*WT/WT*^ mice, the microspheres moved synchronously in a single direction within the lateral ventricle. In contrast, the microspheres moved in several directions indicative of disorganized, turbulent flow in the ventricle of heterozygous *Foxj1*^*WT/CreERT2::GFP*^ mice (Fig. 6a, 6b, Supplementary Videos). The distribution of the direction of movement of individual microspheres in the lateral ventricles of *Foxj1*^*WT/CreERT2::GFP*^ mice was significantly different from that observed in the ventricles of *Foxj1*^*WT/WT*^ mice (*P* < 0.01, Watson’s Two-Sample Test of Homogeneity, Supplementary Fig. 2).

## Discussion

*FOXJ1* is a master regulator of multiciliated cell differentiation (Yu et al. 2008; Jacquet et al. 2009; Mukherjee et al. 2019). The generation of multiciliated cells is required for the proper function of many tissues, including the respiratory tract, brain, and kidney (Terre et al. 2016). Previous work in frogs, zebrafish, and mice revealed a key role for *Foxj1* in determining left–right body symmetry and in forming motile cilia on epithelial cells in the trachea (Brody et al. 2000; Stubbs et al. 2008). Published reports have strongly associated heterozygous *FOXJ1* mutations with defects in mucociliary clearance, situs inversus, and obstructive hydrocephalus in humans, consistent with the known pattern of expression of this transcription factor in multiciliated epithelia. The fact that the loss of function of one allele leads to this constellation of symptoms reveals *FOXJ1* haploinsufficiency as the likely mechanism underlying the autosomal dominant inheritance pattern of this disorder. Here, we report a case of a patient with iNPH who harbors a likely pathogenic heterozygous *FOXJ1* mutation. Interestingly, one of the reports of *FOXJ1*-associated hydrocephalus described a patient with enlargement of the lateral, third, and fourth ventricles, a pattern that is usually associated with communicating hydrocephalus (Wallmeier et al. 2019). The authors suggested that obstruction of the foramina of Luschka and Magendie was responsible for the observed pattern of hydrocephalus, although direct evidence for that was not presented. In light of our finding that heterozygous *FOXJ1* mutation is associated with communicating hydrocephalus in humans and in mice, one must consider the possibility that the patient actually had communicating hydrocephalus. Together, our findings suggest that heterozygous *FOXJ1* mutations can cause either obstructive or communicating forms of hydrocephalus in humans, and they imply the existence of a mechanism contributing to hydrocephalus other than physical obstruction to CSF flow through the ventricular system.

Several reports indicate that heterozygous de novo mutations in *FOXJ1* cause hydrocephalus internus, with or without chronic destructive airway disease (COPD) and randomization of left/right body asymmetry (Wallmeier et al. 2019). The patient whom we describe here presented with typical clinical features of normal pressure hydrocephalus had previously been diagnosed with COPD, and had also been treated for lung cancer. Until now, the majority of published de novo mutations in *FOXJ1* have been loss of function mutations (stop gain codons), and there was also a missense mutation that was related to a case of congenital hydrocephalus (Jin et al. 2020). The R287Q mutation in human *FOXJ1* that we report here is a rare mutation that has a minor allele frequency (MAF) of 2.97e-5 in the general population. We have used three different prediction algorithms to analyze this mutation, and all three indicate that the R287Q mutation probably alters FOXJ1 protein function. Further studies are needed to determine whether this mutation indeed alters FOXJ1 interactions with protein-binding partners, reduces FOXJ1 transcriptional activity, or decreases the expression of *FOXJ1* mRNA or the encoded protein*.*

Studies on multiciliated cells obtained from the respiratory tracts of patients with heterozygous *FOXJ1* mutation have identified defects in motile cilia axonemes as well as a decreased number of motile cilia (Wallmeier et al. 2019). Ependymal cells from the brains of these patients were not examined. However, our studies in mice suggest that the consequences of heterozygous *Foxj1* mutation extend to the multiciliated ependymal cells that line the ventricles of the brain and underly the observed hydrocephalus. We found that mice with decreased expression of *Foxj1* have ependymal motile cilia that display varying defects, including a loss of the typical 9 + 2 microtubule structure and axonemal breaks. These structural changes to motile cilia may be the result of impaired vesicular transport of ciliary proteins required for basal body and axoneme assembly at the apical membrane, a process that is regulated by *Foxj1* (Choksi et al. 2014; Mukherjee et al. 2019; Brody et al. 2000). While the pulsatile bulk flow of CSF is driven by the respiratory and cardiac systems, the complex movement of near-wall CSF is driven by the directional beating of motile cilia (Olstad et al. 2019). Defects in motile cilia structure and function disrupt the near-wall directional movement of CSF and lead to hydrocephalus, albeit via unclear mechanisms (Kumar et al. 2021). We found that mice with heterozygous *Foxj1* mutations display aberrant rotational and planar cell polarity as well as turbulent, disorganized flow in the lateral ventricles.

Traditionally, the polarity of multiciliated ependymal cells is conceptualized in four forms: rotational polarity (i.e., orientation and alignment of the basal bodies), translational polarity (i.e., localization of the basal body patch within the cell), tissue-level or planar cell polarity (i.e., orientation and alignment of the cells on the tissue surface), and apicobasal polarity (i.e., differential specialization of the apical and basolateral surfaces of cells) (Mirzadeh et al. 2010; Wallingford 2010). In addition, our investigation of Feret’s angle suggests that the directional orientation of the basal body patch within the cell represents yet another dimension of cell polarity. The ability of ependymal motile cilia to beat directionally on the ventricular surface and effectively drive directional CSF flow can be affected by disruption of any of these dimensions of cell polarity. In heterozygous *Foxj1* mutant mice, we observed striking differences in planar cell polarity (tissue-level ependymal cell orientation), translational polarity (basal body patch localization within cells), basal body patch orientation (Feret’s angle), and rotational polarity (orientation and alignment of the basal bodies).

The disruption of tissue-level planar cell polarity suggests that *Foxj1* regulates the expression and/or transport of proteins such as VANGL2 that are necessary for the establishment of planar cell polarity. Loss of motile cilia reportedly does not affect translational polarity in ependymal cells (Mirzadeh et al. 2010). Motile cilia are present on the ependymal cells of heterozygous *Foxj1* mice, suggesting that the loss of translational polarity observed in ependymal cells from these mice is the result of a distinct mechanism. *Foxj1* plays a role in directing basal body migration and docking, and impairment of this function could contribute to the loss of translational polarity observed here (Brooks and Wallingford 2014; Boutin et al. 2014). The observed decrease in attachment of the basal bodies to the cytoskeleton in heterozygous *Foxj1* mutant mice supports this hypothesis. Rotational polarity of the basal bodies was also affected in *Foxj1* heterozygotes, indicating that *Foxj1* is involved in the orientation and alignment of basal bodies. Our finding is also supported by several reports that defects in rotational polarity are associated with hydrocephalus development (Ryu et al. 2021; Guirao et al. 2010; Mirzadeh et al. 2010).

Studies of stroke, spinal cord injury, and impaired neurogenesis have suggested that ependymal cells are replaced by GFAP+ astrocytes as a form of gliotic scarring (Carlen et al. 2009; Meletis et al. 2008; Kuo et al. 2006). Heterozygous *Foxj1* mutant mice with hydrocephalus showed increased numbers of GFAP+ cells in the ependymal layer of the lateral ventricles. However, we found that nearly all of these GFAP+ cells strongly express the ependymal cell marker S100β. We further found that many GFAP+ ventricular cells co-stained with two additional ependymal cell markers, αSMA and FOXJ1 (Shah et al. 2018). Because ependymal cells are derived from GFAP+ radial glia, our finding of increased numbers of GFAP+/S100β+ , GFAP+/FOXJ1+ , and GFAP+/αSMA+ cells in the ependymal layer of heterozygous *Foxj1* mutant mice suggests that these are ependymal cells in an immature or intermediate state of differentiation, and not mature GFAP+ astrocytes filling areas of denuded ependyma. Indeed, ependymal cells can adopt an astrocyte-like morphology after stroke (Muthusamy et al. 2018). Additional evidence for intermediate ependymal cell differentiation is supported by our finding of decreased FOXJ1 expression and incomplete development of motile cilia in ependymal cells of heterozygous* Foxj1* mutant mice. In support of the hypothesis that *Foxj1* haploinsufficiency is responsible for persistent GFAP expression in incompletely differentiated ependymal cells, continual *Foxj1* expression has been shown to be necessary to maintain ependymal cell differentiation in adult mice (Abdi et al. 2018).

Genomic sequencing of DNA obtained from patients with hydrocephalus has identified *FOXJ1* as a recurrently mutated gene in a subset of individuals. In each of these cases, the patients presented with evidence of obstructive hydrocephalus (Jin et al. 2020; Wallmeier et al. 2019; Shapiro et al. 2021). However, we report here a case of communicating hydrocephalus occurring in the context of heterozygous *FOXJ1* mutation. In heterozygous *Foxj1* mutant mice, we found that the aqueduct remained patent with no evidence for obstruction to CSF flow. In mice, the *Foxj1*-associated hydrocephalus appears to result from incomplete differentiation of multiciliated ependymal cells in the context of *Foxj1* haploinsufficiency, leading to motile cilia dysfunction, disturbed CSF flow, and hydrocephalus. Our findings suggest that the CSF flow obstruction observed in humans harboring heterozygous *FOXJ1* mutations is not the primary cause of hydrocephalus but develops secondarily. Work on zebrafish larvae has shown that the ventricular ducts rely on the flow of CSF to maintain patency (Olstad et al. 2019). If motile cilia function is impaired (as observed in mice and in humans with heterozygous *FOXJ1* mutations), the disruption to CSF flow could promote duct closure. Indeed, expansion of the ventricular system alone can lead to reversible obstruction of the aqueduct in human congenital hydrocephalus (Duy et al. 2022). However, alternative hypotheses may also be considered. For example, it is possible that different *FOXJ1* mutations have different effects, leading to communicating or obstructive hydrocephalus by distinct and unrelated mechanisms. Taken together, our findings shed new light on the possible mechanisms by which heterozygous *FOXJ1* mutations cause hydrocephalus in humans and in mice.

### Supplementary Information

Below is the link to the electronic supplementary material.

Supplementary file1 (AVI 47248 kb)

Supplementary file2 (AVI 26964 kb)

Supplementary file3 (PDF 769 kb)

## Data Availability

All data needed to evaluate the conclusions are presented in the manuscript.

## Abbreviations

BB: Basal body
CSF: Cerebrospinal fluid
GFAP: Glial fibrillary acidic protein
iNPH: Idiopathic normal pressure hydrocephalus
MAF: Minor allele frequency
PCP: Planar cell polarity
SEM: Scanning electron microscopy
αSMA: Smooth muscle actin alpha
TEM: Transmission electron microscopy
WT: Wild type
