# Supplementary material for: Heterozygous FOXJ1 Mutations Cause Incomplete Ependymal Cell Differentiation and Communicating Hydrocephalus
Source: Cell Mol Neurobiol. 2023 Aug 24;43(8):4103–16. doi: 10.1007/s10571-023-01398-6 (PMC10661798; doi:10.1007/s10571-023-01398-6)
Supplement: Supplementary file 3 — Supplementary file3 (PDF 769 kb) [file 10571_2023_1398_MOESM3_ESM.pdf]

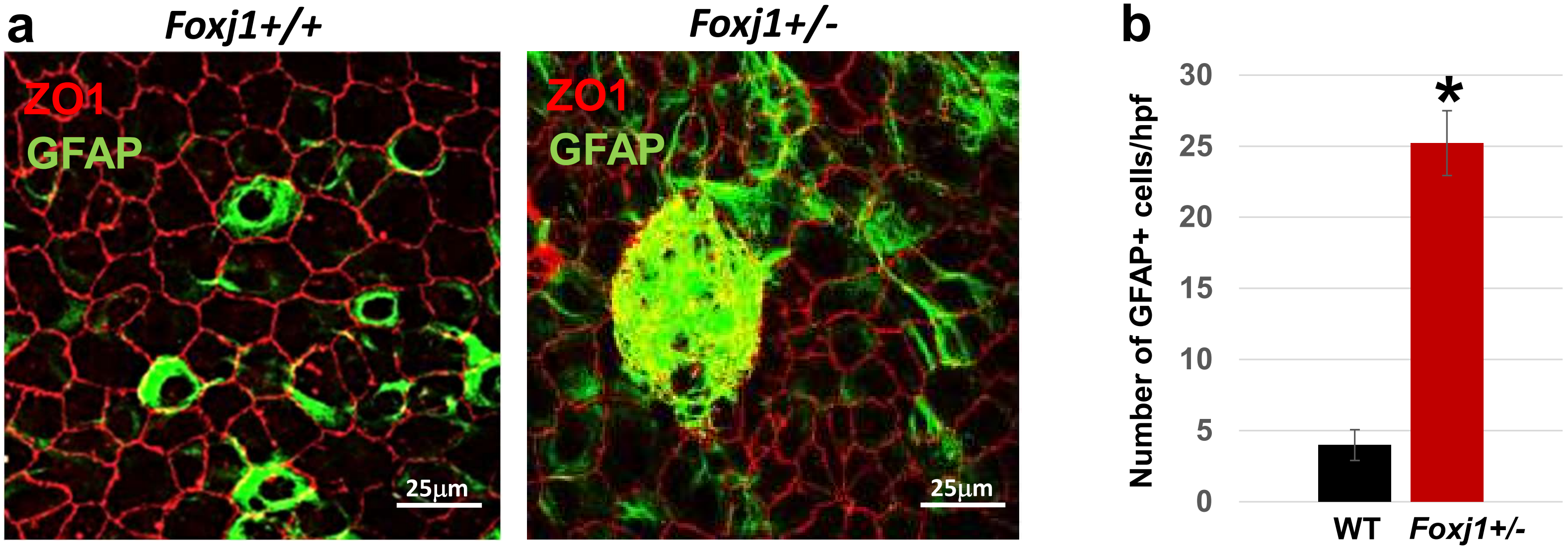

(a) Immunohistochemistry of the lateral ventricular wall for GFAP (green) and ZO-1 (red).
(b) Quantification of GFAP positive cells observed along the lateral ventricular wall.

Supplementary Figure 2

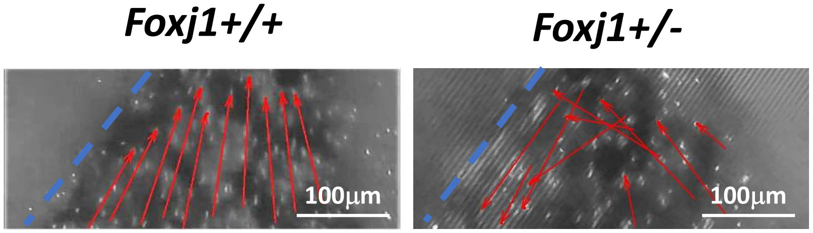

Quantification of CSF dynamics. Dotted blue line indicates edge of lateral ventricular wall explant; red arrows indicate the direction of movement of microspheres.

Angular direction of movement of microspheres

Foxj1+/+	Foxj1+/-
9.33	-203.3
27.37	-185.55
41.19	-176.07
20.23	-174.89
48.53	43.59
32.19	68.12
39.26	75.71
24.7	80.37
17.98	90.45
6.57	111.88

P*<0.01, *Watson's Two-Sample Test of Homogeneity
